# Thermo-Mechanical Behavior of Novel EPDM Foams Containing a Phase Change Material for Thermal Energy Storage Applications

**DOI:** 10.3390/polym14194058

**Published:** 2022-09-27

**Authors:** Marica Bianchi, Francesco Valentini, Giulia Fredi, Andrea Dorigato, Alessandro Pegoretti

**Affiliations:** INSTM Research Unit, Department of Industrial Engineering, University of Trento, Via Sommarive 9, 38123 Trento, Italy

**Keywords:** EPDM, rubber, foams, thermal energy storage, PCM, paraffin

## Abstract

In this paper Ethylene Propylene Diene Monomer rubber (EPDM) foams were filled with different amounts of paraffin, a common phase change material (PCM) having a melting temperature at about 70 °C, to develop novel rubber foams with thermal energy storage (TES) capabilities. Samples were prepared by melt compounding and hot pressing, and the effects of three foaming methods were investigated. In particular, two series of samples were produced through conventional foaming techniques, involving physical (Micropearl^®^ F82, MP, Lehvoss Italia s.r.l. Saronno, Italia) and chemical (Hostatron^®^ P0168, H, Clariant GmbH, Ahrensburg, Germany) blowing agents, while the salt leaching method was adopted to produce another series of foams. Scanning electron microscopy (SEM) and density measurements showed that MP led to the formation of a closed-cell porosity, while a mixed closed-cell/open-cell morphology was detected for the H foamed samples. On the other hand, foams produced through salt leaching were mainly characterized by an open-cell porosity. The qualitative analysis of paraffin leakage revealed that at 90 °C only the foams produced through salt leaching suffered from significant PCM leakage. Consequently, the thermo-mechanical properties were investigated only in samples produced with H and MP. Differential Scanning Calorimetry (DSC) analysis revealed that EPDM/paraffin foams were endowed by good TES properties, especially at higher PCM contents (up to 145 J/g with a paraffin amount of 60 wt%). Tensile and compressive tests demonstrated the addition of the PCM increased the stiffness at 25 °C, while the opposite effect was observed above the melting temperature of paraffin. These results suggest that the EPDM foams produced with H and MP show an interesting potential for thermal management of electronic devices.

## 1. Introduction

The term “global warming” refers to the rise in the average surface temperature of the Earth caused by the increase in the concentration of greenhouse gases (GHGs) and it is becoming one of the most urgent global problems [1]. According to Intergovernmental Panel on Climate Change (IPCC) of 2018, the global temperature has risen by 1.0 °C in the past 150 years and, if warming continues at the current rate, it is expected to reach 1.5 °C, or even 2.0 °C, between 2030 and 2052 [2]. It is clear that actions are needed to limit irreversible consequences on ecosystems and human beings [3]. Several studies have underlined that a decisive contribution to resolve global warming would be the limitation of GHGs emissions and, consequently, a reduction of the use of energy that represents by far the largest source of such emissions from human activities [3]. To reach this target, environmental laws have grown significantly over the last years, stimulating the research on innovative systems and techniques capable to slow down the temperature growth rate, thereby limiting global warming. In this scenario, one of the most promising research fields is that of the energy storage systems. The working principle of these systems consists in the accumulation of different forms of energy when available, to use at a later time when and where needed [4,5,6].

Among the several methods of energy storage, one of the most widely investigated is thermal energy storage (TES). TES allows excess or loss of thermal energy to be temporarily stored in a storage medium and used at a later time for heating/cooling applications or power generation [7]. Three categories of TES systems can be identified: latent heat, sensible heat, and thermo-chemical heat storage. Among these, latent heat storage becomes of great interest when a large amount of thermal energy needs to be accumulated. In these systems, the heat storage/release mechanism is the result of a phase change (typically a solid-liquid transition) that occurs in a specific narrow temperature range in a so-called phase change material (PCM), characterized by latent heat at the transition temperature [8,9]. Typical PCMs are, for example, paraffin waxes, fatty acids, poly(ethylene glycol)s, salts, and salt hydrates. Paraffin waxes are relatively cheap organic PCMs, known for their high heat of fusion, chemical stability, and availability in a large melting temperature range [10]. Due to their unique properties, they have been widely studied for thermal regulation of smart fabrics, sportswear, buildings, and electronic devices [6,11,12,13,14,15,16,17,18,19,20,21,22,23,24,25,26,27,28,29]. To avoid leakage during the melting process, their confinement can be performed by encapsulation or shape-stabilization. More specifically, an easiest and inexpensive type of shape-stabilization consists in mixing the PCM with a supporting material, able to host a large amount of PCM and to avoid its leakage at the molten state [9,10].

One of the potential candidates to perform this task is the family of elastomeric foams, also known as cellular rubbers. Despite their potential as polymeric hosting material [4,30], very few works in the literature have dealt with the investigation of these materials as a shape stabilizer. Elastomeric foams are soft and elastic and characterized by low thermal conductivity values, high resistance to moisture (if a closed-cell morphology is realized), and good fire performance. All these features make them suitable for several applications, in particular for thermal and acoustic insulation [31]. In the literature, various types of rubber foams are reported, based on natural rubber (NR), polyisoprene rubber (IR), polychloroprene rubber (CR), styrene-butadiene rubber (SBR), ethylene-vinyl acetate (EVA), isobutylene rubber (IIR), acrylonitrile-butadiene rubber (NBR), polyurethane (PU), chlorinated polyethylene rubber (CPE), silicone rubber (SR), ethylene propylene diene monomer rubber (EPDM), etc. [32,33,34,35,36,37,38,39]. Due to their excellent protection against polar fluids, polar solvents, alcohols, and ammonia, high tear and abrasion resistance, and high weather, ozone, and oxidation resistance, EPDM foams have received great attention for outdoor applications such as building profiles, wire materials, electronic components, roofing sheets, sporting goods, and automotive sealing systems [32,40].

The foaming process for the production of elastomeric foams is generally carried out by incorporating chemical or physical blowing agents that, with increasing temperature, develop gases responsible for the polymer expansion [41]. More specifically, physical blowing agents are fluids that provide gases by changing their physical state, while chemical blowing agents are compounds that evolve gases with their thermal degradation [41]. Despite the benefits of such blowing agents, like a broad operating window and a generation of a fine cell size, there are also negative aspects that have to be considered. Many blowing agents, in particular the chemical ones, are hazardous for health, cannot be used in the EU, and have a strong impact on the environment, contributing to the damage of Earth’s ozone layer [42]. Hence, alternative methods to the traditional foaming process have been recently proposed. Among these, salt leaching technique seems to be a valid alternative. It involves the addition of two water-soluble particles (such as sodium chloride and polyethylene glycol (PEG)) into the polymer matrix and the subsequent dissolution of these compounds in hot water, leading to the generation of a cellular network with open porosity throughout the polymer matrix [43,44,45]. Sodium chloride has a fundamental role since salt granulometry controls the pore dimensions of the foam. On the other hand, PEG is responsible for generating a pore network in the cellular material.

Based on these considerations, this work focuses on the thermo-mechanical characterization of EPDM/paraffin foams, produced both through the conventional foaming techniques, involving physical and chemical blowing agents, and both through the salt leaching method. With the intention to use these foams for the thermal regulation of electronic devices, a paraffin wax having a melting temperature of 70 °C has been selected. This work aims at investigating the effect of the production process and of the paraffin amount on the thermo-mechanical behavior of the resulting foams, in view of their future application as thermo-regulating TES systems.

## 2. Materials and Methods

### 2.1. Materials

Vistalon^®^ 2504 EPDM rubber is an amorphous terpolymer containing 58 wt% of ethylene and 4.7 wt% of ethylidene norbornene, purchased from Exxon Mobil (Irving, TX, USA). It is characterized by a broad molecular weight distribution and a Mooney viscosity (ML 1 + 4, 125 °C) of 25 MU. The chemicals employed in the vulcanization were supplied by Rhein Chemie (Cologne, Germany), and consisted of sulfur as a vulcanizing agent, zinc oxide and stearic acid as curing activators. Carbon Black N550, obtained from Omsk carbon group (Prescott, ON, Canada), was used as a reinforcing filler. Process accelerators tetramethylthiuram disulfide (TMTD) and zinc dibutyl dithiocarbamate (ZDBC), purchased from Vibiplast srl (Castano Primo, Italy), were provided separately and were added during the compounding process. The composition (in phr) of the elastomeric compound used for the preparation of the samples is given in Table 1.

Polymeric granules of poly (ethylene glycol) (PEG) with a molecular weight of 2000 Da were acquired from Alfa Aesar (Kandel, Germany). Sodium Chloride (NaCl) having a density of 2.16 g/cm^3^ was purchased on the market. In order to obtain a salt granulometry comprised between 63 and 80 µm, NaCl was milled and then sieved using 230 and 200 mesh sieves. The choice of the salt grain size was dictated by the results of our previous studies [43]. Before use, NaCl was stored in an oven at 40 °C for at least 24 h to avoid humidity absorption.

Two foaming agents were adopted for the expansion of EPDM rubber: Hostatron^®^ P0168 (H), acquired from Clariant GmbH (Ahrensburg, Germany) and Micropearl^®^ F82 (MP), supplied by Lehvoss Italia s.r.l. (Saronno, Italia). Micropearl F82 consists of microspheres having a size ranging from 20 to 30 µm and containing a low-boiling-point hydrocarbon (iso-pentane) that, increasing the temperature above 115–120 °C, starts expanding due to the vaporization of the hydrocarbon. Hostatron P0168 is a mixture of sodium, calcium, and potassium bicarbonates. The expansion occurs through the decomposition of the bicarbonates taking place at 150 °C, with evolution of carbonates, CO_2_, and vapor as reaction products.

Rubitherm^®^ RT70HC is a paraffin characterized by a melting point of 70 °C and a melting enthalpy of 260 J/g. It was purchased from Rubitherm GmbH (Berlin, Germany). This phase change material was selected to store/release thermal energy in a temperature interval close to 70 °C since the aim of the work was to produce a thermal management system for electronic devices.

### 2.2. Sample Preparation

Three types of EPDM foams have been prepared and characterized. In particular, two series of expanded rubbers were produced using H and MP foaming agents, while the salt leaching method was adopted to produce the third series of foams. Furthermore, for each of these series of cellular rubbers, the neat foam (constituted only by EPDM rubber) and EPDM foams containing different amounts of paraffin wax were analyzed. The PCM content in the samples was chosen equal to 20, 40, and 60 wt%, with respect to the EPDM compound to investigate the effect of a low, medium, and high amount of PCM on the thermo-mechanical and TES properties of the produced foams. In order to compare the properties of foamed and unfoamed samples, a sample of bulk EPDM rubber was also produced. The samples were prepared by melt compounding in an internal mixer (Thermo Haake Rheomix 600), equipped with counter-rotating rotors. Depending on the final product, the compounding temperature was kept at temperatures listed in Table 2 and maintained for the whole mixing process. The rotor speed was set at 50 rpm.

At first, the EPDM compound was prepared by adding Vistalon^®^ 2504, sulfur, carbon black, stearic acid, and zinc oxide into the mixer and the mixture was compounded for 5 min. Then, the accelerators TMTD and ZDBC were added and mixed for further 5 min. After that, the procedure was different for unfoamed and foamed samples, and for neat and PCM-containing samples.

#### 2.2.1. Production of Neat Unfoamed and Foamed Samples

- To produce the non-expanded EPDM sample, after a total compounding time of 10 min, the material was vulcanized under a hydraulic press at a pressure of 8 bar and a temperature of 170 °C in a close mold. The dimensions of the mold were 110 × 110 × 5 mm^3^.
- To produce the neat EPDM foams using H and MP, after 10 min of compounding, the foaming agent was added and mixed for 5 additional minutes. The vulcanization of the material was then carried out in a close mold at a pressure of 1 bar and a temperature of 170 °C. After 10 min, the pressure was released to allow the expansion of the material, and the samples were left for further 10 min to guarantee the completion of the vulcanization process. The dimensions of the mold were 110 × 110 × 5 mm^3^ and 110 × 110 × 10 mm^3^ for foams produced with H and MP, respectively. Due to a greater foaming tendency observed in foams produced with MP in preliminary tests, a higher height of the mold was selected for this kind of expanded rubbers.
- To produce neat foams through salt leaching, NaCl and PEG were gradually added to the mixer in order to obtain a homogeneous compound. To assure a uniform distribution of PEG and NaCl, the compound was then milled by means of an IKA M20 Universal mill (IKA, Germany) and fed again in the mixer for a mixing time of 5 min. The vulcanization of the final compound was carried out in a closed mold having dimensions of 110 × 110 × 5 mm^3^, under a pressure of 1 bar and at a temperature of 170 °C for 20 min. Finally, NaCl and PEG were removed in demineralized water at a temperature of 100 °C for 3 h, leading to the formation of a porous structure in the EPDM matrix. The samples were then dried overnight in an oven at 60 °C.

#### 2.2.2. Production of EPDM/Paraffin Foams

- To produce EPDM/paraffin foams with H and MP, the same procedure of the corresponding neat samples was followed, with the only difference that paraffin was gradually fed in the mixer after the addition of the foaming agent and compounded for 20 min, to obtain a uniform distribution of paraffin in the compound.
- To prepare EPDM/paraffin foams through salt leaching method, once the corresponding neat foam was produced, the addition of paraffin was carried out by pouring the molten PCM at 90 °C on the surface of the sample. With the aid of a spoon, paraffin was distributed on the surface of the foam before its solidification took place. Finally, the sample was maintained for 20 min in an oven at 90 °C, to improve impregnation.

The sample preparation resulted in square sheets with in-plane dimensions of 110 × 110 mm^2^ and a thickness variable in the range 5–10 mm, depending on the type of foaming procedure. The composition (in phr) of the prepared foams along with their codes is reported in Table 3. The foaming agent contents were selected based on the results of previous works, in which it was demonstrated that such contents were optimum for foams preparation [4,29]. The sample named EPDM refers to the non-expanded EPDM rubber. The code of the samples is composed by the term EPDM followed by a letter/s that identify the production method (H: Hostatron foaming agent, MP: Micropearl foaming agent, SL: salt leaching method), the letter P, which refers to paraffin (in foams containing the PCM), and finally a number that coincides with the weight percentage of paraffin in the sample.

### 2.3. Characterization

#### 2.3.1. Scanning Electron Microscopy (SEM)

The microstructure of the prepared foams was investigated by means of a field emission scanning electron microscopy (FESEM) AG-SUPRA 40 (Carl Zeiss, Oberkochen, Germany), operating at an accelerating voltage of 2.5 kV. The samples were soaked in liquid nitrogen for 2 h and then cryofractured. The fracture surface was analyzed after Pd-Pt sputtering to provide enhanced electrical conductivity. For each type of foam, 15 measurements of the pore diameter were performed using ImageJ^®^ software(NIH, Bethesda, MD, USA). In the case of cells with an elliptical section, the major axis of the ellipse was considered to perform the measurements.

#### 2.3.2. Density Measurements

The apparent density (*ρ_app_*) of the produced EPDM foams was determined by helium pycnometry. Tests were conducted using a gas displacement AccuPycII 1330 pycnometer (Micrometrics Instrument Corporation, Norcross, GA, USA) on specimens of approx. 0.2 g and at a temperature of 23.0 °C in helium. For each sample, 60 replicate measures were performed. Geometrical density measurements (*ρ_geom_*) were determined on 10 square specimens, derived from each EPDM sample. The mass of the foams was measured using a Gibertini E42 balance (sensitivity 10^−4^ g) while their dimensions by means of a caliper (sensitivity 0.01 mm). The *ρ_geom_* was finally calculated by dividing the mass over the total volume, inclusive of bulk material, closed and open porosity. According to ASTM D6226 standard, it was possible to determine the total porosity (*P_tot_*), the fraction of open porosity (*OP*) and closed porosity (*CP*) according to Equations (1)–(3), respectively.

(1) $$P_{tot}=\left(1-\frac{\rho_{geom}}{\rho_{bulk}}\right)\times 100$$

(2) $$OP=\left(1-\frac{\rho_{geom}}{\rho_{app}}\right)\times 100$$

(3) $$CP=P_{tot}-OP$$

where *ρ_bulk_* is the density of the material without porosity (i.e., 1.042 g/cm^3^ for the unfoamed EPDM sample, as reported in our previous work on this EPDM system [42]).

#### 2.3.3. Qualitative Evaluation of Paraffin Leakage

In order to investigate the capability of the foams in retaining paraffin at 90 °C, i.e., above the melting temperature of the PCM, a simple and qualitative leakage test was carried out. Rectangular specimens (length 100 mm, width 10 mm, thickness 5 mm) were cut from the produced EPDM/paraffin foams and placed over a sheet of tissue paper. The specimens, together with the sheet, were then maintained for 1 h in an oven at 90 °C. After the set time, the specimens were removed from the paper sheet and the leakage of paraffin was investigated by visual inspection.

#### 2.3.4. Rheology

In order to investigate the effect of the foaming agent on the viscosity of the uncured mixture, dynamic rheological tests were carried out by means of a DHR-2 Rheometer (TA Instrument, New Castle, DE, USA) in plate-plate configuration and isothermal conditions, setting a gap distance of 1 mm. Disc samples (diameter 25 mm, thickness 1 mm) were selected for the measurements. The test was conducted in frequency sweep mode, at 100 °C and at a maximum shear strain of 1%. Complex viscosity (η*) was evaluated in the angular frequency range 0.1–1000 rad/s and the test was conducted on four compositions: EPDM_H, EPDM_MP, EPDM_H_P40, EPDM_MP_P40. One specimen was tested for each formulation.

#### 2.3.5. Thermogravimetric Analysis (TGA)

Thermogravimetric analysis (TGA) was performed through a Mettler TG50 thermobalance (Mettler-Toledo GmbH, Schwerzenbach, Switzerland) in order to investigate the influence of the foaming process and the paraffin fraction on the degradation resistance of the materials. Samples were tested at a heating rate of 10 °C/min from 25 °C up to 700 °C under a nitrogen flow of 10 mL/min. One specimen for each composition was tested. The temperature associated with a mass loss of 5% was evaluated (T_5%_). In addition, from the first derivative of the thermogravimetric curve (DTG), the temperatures corresponding to the maximum mass loss rate of paraffin (T_peak1_) and of the EPDM matrix (T_peak2_) were determined. Finally, in order to evaluate the effective PCM content inside the produced foams (PCM_eff_), the mass loss at 400 °C was determined.

#### 2.3.6. Differential Scanning Calorimetry (DSC)

Differential scanning calorimetry (DSC) tests were performed on the prepared foams by means of a Mettler DSC30 calorimeter (Mettler Toledo GmbH, Schwerzenbach, Switzerland). One specimen was tested for each composition, with a mass of approximately 30 mg, weighted using a balance Gilbertini E42 (sensitivity 10^−4^ g). The specimens were subjected to the following thermal-ramps: (I) first heating scan from 30 °C to 100 °C at 1 °C/min, (II) cooling scan from 100 °C to 30 °C at 1 °C/min, (III) final heating scan from 30 °C to 100 °C at 1 °C/min. Nitrogen was used as the purge gas at a flow of 100 mL/min. DSC analysis allowed the measurement of the melting temperature (T_m_) and of the crystallization temperature (T_c_) of paraffin, and the corresponding specific enthalpy of melting (Δ*H_m_*) and crystallization (Δ*H_c_*). Moreover, as it was done in our previous work [4], the effective paraffin content in the specimens was determined by assuming a melting and crystallization efficiency of paraffin equal to 100%, i.e., assuming that the melting and crystallization enthalpy of paraffin does not decrease when the paraffin is embedded in EPDM. In particular, the effective paraffin content in the first heating scan ($PCM_{m1}^{eff}$), the cooling scan ($PCM_{c}^{eff}$), and the second heating scan ($PCM_{m2}^{eff}$) was determined as the ratio between the specific enthalpy of the samples and the corresponding specific enthalpy values of the neat paraffin, as shown in Equations (4)–(6), respectively.

(4) $$PCM_{m1}^{eff}=\left(\frac{\Delta H_{m1}}{\Delta H_{m1PCM}}\right)\times 100$$

(5) $$PCM_{c}^{eff}=\left(\frac{\Delta H_{c}}{\Delta H_{cPCM}}\right)\times 100$$

(6) $$PCM_{m2}^{eff}=\left(\frac{\Delta H_{m2}}{\Delta H_{m2PCM}}\right)\times 100$$

where Δ*H_m_*_1*PCM*_, Δ*H_cPCM_*, Δ*H_m_*_2*PCM*_ are the specific enthalpy values associated to the melting during the first heating scan, the crystallization in the cooling stage, and melting during the second heating scan of the neat paraffin, respectively.

The retention of the thermal energy storage/release capability was assessed through cyclic DSC tests on formulations containing the maximum paraffin concentration. The tests were performed between 0 °C and 120 °C at 10 °C/min, under a nitrogen flow of 100 mL/min. A total of 20 cycles were carried out for each formulation, with one cycle constituted of one heating and one cooling ramp.

#### 2.3.7. Thermal Conductivity Measurements

Thermal conductivity measurements were carried out by means of a heat flow meter apparatus (HFM 446 Lambda, NETZSCH Instrument, Selb, Germany) on square EPDM and EPDM/paraffin samples. Test specimens had dimensions 110 × 110 × 5 mm^3^ for bulk EPDM and foams produced through salt leaching and with H foaming agent, while 110 × 110 × 10 mm^3^ for foams produced with MP foaming agent. One specimen was tested for each composition. Measurements were performed at a mean temperature of 30 °C with a temperature difference between the hot and cold plates of 20 °C.

#### 2.3.8. Quasi-Static Tensile Tests

Tensile properties under quasi-static conditions were measured by means of an Instron 5969 tensile testing machine, equipped with a load cell of 10 kN and operating at a cross-head speed of 100 mm/min. The tests were performed on ISO 527 type 1BA specimens at 25 °C and 90 °C, i.e., below and above the melting point of paraffin, respectively. The elastic modulus normalized by the geometrical density of each sample (E*) was measured as a secant value between two strain levels (approx. 0.1 and 0.2 mm/mm) in the linear stress-strain region. Moreover, the tensile strength at break normalized by geometrical density (σ_b_*) and the corresponding strain level (ε_b_) were determined. Five specimens were tested for each composition and temperature.

#### 2.3.9. Quasi-Static Compression Tests

Compressive properties under quasi-static conditions were measured on square specimens obtained from the produced foams. The dimensions of the test specimens were selected considering the thickness of the foams, in order to comply with the aspect ratio defined by the ISO 844 standard. In particular, test specimens had dimensions 15 × 15 × 5 mm^3^ for bulk EPDM and foams produced through salt leaching method and with H, and 21 × 21 × 10 mm^3^ for foams produced with MP. The tests were performed below (25 °C) and above (90 °C) the melting point of paraffin by means of an Instron 5969 tensile testing machine, equipped with a load cell of 10 kN. The cross-head speed was set at 0.5 mm/min and, for foams produced with MP, at 1.0 mm/min. The compressive modulus normalized by the geometrical density of each sample (E_c_*) was measured as a secant value between the strain levels of 0.15 and 0.3 mm/mm. Moreover, the deformation of the samples corresponding to a stress equal to 5 MPa (ε_5_) and the stress values (normalized by the geometrical density of each sample) corresponding to a deformation of 0.6 mm/mm (σ_0.6_) were evaluated. Five specimens were tested for each composition and temperature.

#### 2.3.10. Shore A

Shore A hardness measurements were performed by using a Hilderbrand Durometer (Prüf-und Meßtechnik GmbH, Wendlingen am Neckar, Germany) following the ASTM D2240 standard. Square specimens 15 mm wide and 5 mm thick were tested, after pressing an indenter against the specimen for a time equal to 10 s. The load level was set at 488 g. The tests were performed at 25 °C and 90 °C, i.e., below and above the melting point of paraffin, respectively. Five specimens were tested for each composition and temperature.

## 3. Results and Discussion

### 3.1. SEM

**Figure 1:**
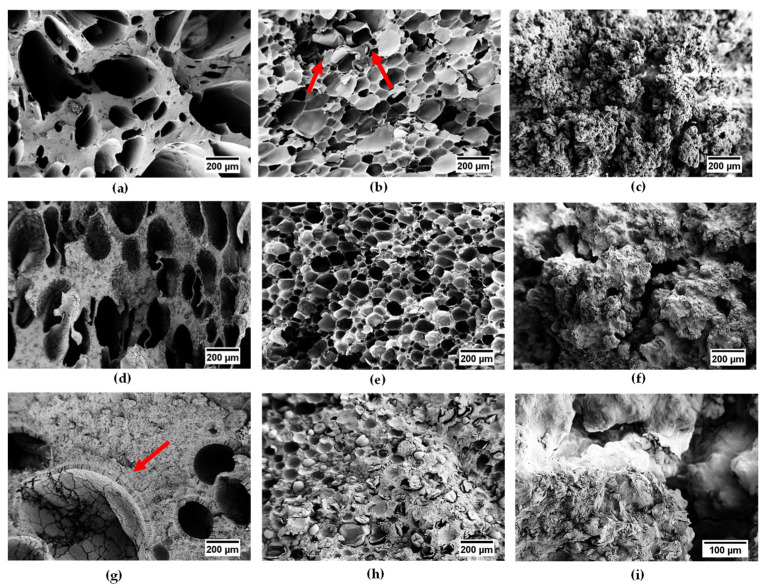
SEM micrographs at different magnification of the cryofracture surface of the prepared foams. (**a**) EPDM_H, (**b**) EPDM_MP, (**c**) EPDM_SL, (**d**) EPDM_H_P20, (**e**) EPDM_MP_P20, (**f**) EPDM_SL_P20, (**g**) EPDM_H_P60, (**h**) EPDM_MP_P60, (**i**) EPDM_SL_P60. The red arrow in Figure 1b indicates the microcapsules containing the physical blowing agent, while in Figure 1g the arrow shows the presence of a peculiar morphology.

SEM observations were performed in order to investigate the morphological features of the produced foams and the effect of the production process and paraffin addition on foams morphology. Figure 1a–i report the micrographs of the cryofracture surface of the produced samples at different magnifications, while in Table 4 the average diameter of the pores is reported.

Analyzing Figure 1a–c, it is possible to highlight that the production process and foaming agent strongly affect the morphology of the foams. In the case of EPDM_H, the sample shows both an open and closed porosity, characterized by a wide pore dimension distribution and different cell shapes. This is confirmed by data listed in Table 4: an average pores size of 271 µm and a standard deviation of 150 µm have been determined for this sample. On the other hand, the use of MP as a foaming agent leads to the development of a closed spherical porosity, with homogeneous and quite uniform pore size distribution, having a mean dimension of approximately 110 µm. As indicated by red arrows in Figure 1b, microcapsules containing the physical blowing agent (i.e., iso-pentane) are still visible inside some broken cells. Completely different is the scenario observed for samples produced via salt leaching. The porosity is open, highly interconnected, and cell boundaries cannot be distinguished. With respect to the previous cases, the foam is characterized by smaller pores.

Micrographs in Figure 1d–f show the produced foams with 20 wt% of paraffin. Comparing their morphological features with the corresponding neat foams, some differences can be highlighted. EPDM_H_P20 sample (Figure 1d) is characterized by a more homogeneous and uniform size distribution. This could be correlated to the presence of paraffin, acting as a plasticizer and favoring the foaming process. On the other hand, the PCM seems to make the rubber expansion more difficult in EPDM_MP_P20 sample. This could be deduced looking at the dimension and geometry of the pores and analyzing numerical values reported in Table 4. The cells appear not so spherical as in the neat foam and with a broader size distribution. The contrasting action exerted by paraffin could be probably due to the different ways it distributes inside the EPDM matrix in the two type of foams. However, further investigations are planned to confirm this hypothesis.

Finally, micrographs in Figure 1g–i show the produced foams with 60 wt% of PCM. Particular attention should be paid to EPDM_H_P60 sample, where a peculiar morphology can be observed. A compact layer of EPDM matrix is sandwiched between two layers of paraffin: one distributed in a lamellar form in the internal surface of the pores and the second one constituting the walls of the foam. This is another clear evidence of the immiscibility of the two phases. Considering the average diameter measured for all foams produced with MP, it can be observed that it progressively reduces with increasing the paraffin content. As it was previously explained, paraffin probably tends to make the foaming process more difficult in these kinds of foams. Unfortunately, for EPDM_SL_P60 sample, it was not possible to determine an average pore dimension with the acquired micrographs.

### 3.2. Density Measurements

To correlate the morphology of the produced foams with their porosity values, density measurements were performed, which allowed the determination of the relative amount of open, closed, and total porosity. The main results are listed in Table 5.

The density measurements highlight that MP foaming agent leads mainly to the formation of closed-cell porosity and lower density values with respect to those obtained with H or with the salt leaching method. This behavior is due to the fact that MP is constituted by thermo-expandable microcapsules containing low boiling point hydrocarbons. When the microcapsules are heated, the shells are softened and expanded with the pressure of gasified hydrocarbon, leading consequently to a closed porosity. Moreover, MP foaming agent leads to the highest value of total porosity. In the case of H foamed rubbers, the expansion is due to the production of CO_2_ and vapor that lead to the formation of porosity with either open or closed cells. Finally, foams produced through the salt leaching method are mainly characterized by an open-cell porosity. In fact, in these samples *ρ_app_* values are much higher than *ρ_geom_* and total porosity can be assumed equal to the open one at all the PCM loadings. As it is possible to notice, *ρ_bulk_* values do not present a standard deviation. It should be reminded that *ρ_bulk_* corresponds to the theoretical density of the samples assuming that no porosity is present. For all the prepared foams, a decrease in the total porosity with increasing paraffin amount can be observed. This could be probably related to the paraffin that tends to hinder the expansion ability of the samples, thereby impairing the microcapsule expansion (in the case of MP foaming agent) and the release of gases (in the case of H foaming agent). The highest reduction in total porosity, moving from the neat EPDM foam to the sample containing 60 wt% of paraffin, is registered in foams produced through salt leaching. In this case, *P_tot_* drops from 64.9% to 25.3%.

### 3.3. Qualitative Analysis of Paraffin Leakage

**Figure 2:**
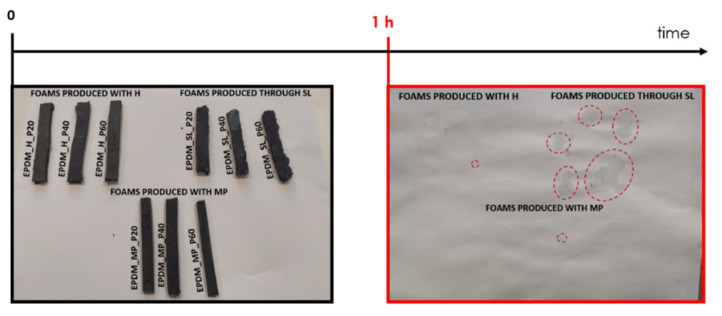
Results of the leakage tests at 90 °C on the prepared paraffin-filled foams. Rectangular specimens were place on a sheet of paper and maintained for 1 h at 90 °C. After the set time, the specimens were removed and eventual leakage of paraffin was investigated.

In order to investigate the capability of the foams to retain paraffin above the melting temperature of the PCM, a simple and qualitative leakage analysis was carried out, and the results of these tests are reported in Figure 2.

A significant leakage of paraffin is observed in all foams produced via salt leaching (i.e., EPDM_SL_P20, EPDM_SL_P40, EPDM_SL_P60), while in the other cases, just little losses from foams with maximum PCM content can be detected. This behavior is probably associated to the different distribution of paraffin within the foams and the different porosity generated by the three foaming methods. In fact, while in samples produced with MP and H the PCM is distributed preferentially inside the foam walls, in foams produced through salt leaching the impregnation of the corresponding neat sample with liquid PCM leads to a stratified system, having an external coating of pure paraffin and an internal layer rich in EPDM rubber, as evidenced in SEM micrographs (see Figure 1f). This stratified structure could explain the loss of PCM found in these foams. Since a significant paraffin leakage would strongly limit their application in electronic devices, it was not considered appropriate to continue the characterization of the samples produced through this methodology. Consequently, the thermo-mechanical properties were studied only in foams produced with H and MP foaming agents. In the future, it would be interesting to investigate if the molecular weight of the PCM could affect the capability of this kind of foams in retaining paraffin. As it was observed in our previous work [4], the impregnation of neat EPDM foams with a paraffin wax having a melting point of 21 °C led to EPDM/paraffin foams characterized by limited PCM loss. Since the production process of the foams was the same, this experimental evidence suggests that the melting point of the PCM, and so its molecular weight, could be responsible for a different leakage tendency.

### 3.4. TGA

**Figure 3:**
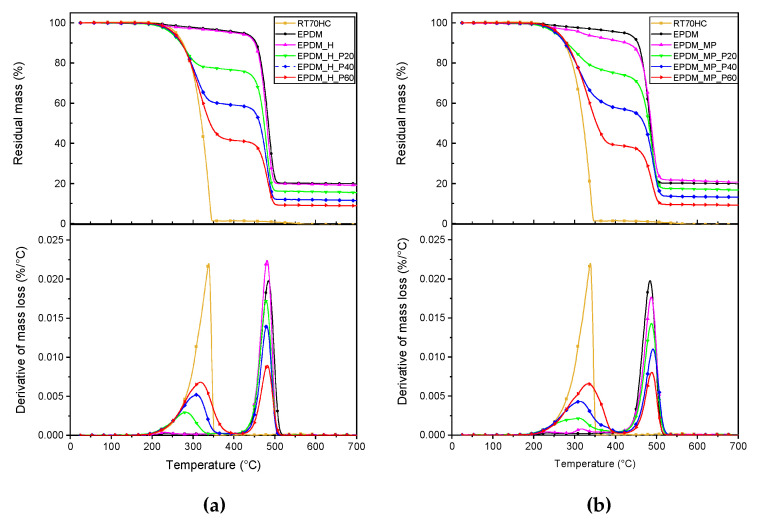
TGA thermograms (residual mass and mass loss derivative as a function of temperature) of bulk EPDM sample, neat paraffin (RT70HC), and foams produced with (**a**) H and (**b**) MP foaming agents.

Thermogravimetric tests were carried out in order to investigate the degradation behavior of the prepared foams and to explore the influence of paraffin amount and production process on their degradation resistance. Thermogravimetric curves of the samples produced with H and MP, along with the corresponding derivative curves, are presented in Figure 3a–b, while the most significant results are reported in Table 6.

Focusing at first on the thermograms of neat unfoamed and foamed EPDM samples reported in Figure 3a,b, it can be noticed that the degradation process of the foamed samples is similar to that of the unexpanded rubber. A weight loss of approx. 5% is observed in the temperature range 317 °C to 420 °C, corresponding to the evaporation of low molecular weight plasticizer and oils used in rubber production. Degradation of EPDM rubber takes place in one single step, starting at around 250 °C and showing a maximum degradation rate at approximately 483 °C in all neat samples. A plateau in the TGA curves above 500 °C can be noticed, highlighting that no significant mass loss in nitrogen atmosphere occurs in the range 500–700 °C. For the residue at 700 °C (m_700_), related to not-degraded carbon black, the bulk EPDM, EPDM_H, and EPDM_MP samples show a similar mass residue of about 20%. Neat paraffin (RT70HC) also degrades in one single step at a temperature around 338 °C and, looking at m_700_ values, the PCM completely degrades without leaving any solid residue.

Moving to TGA curves related to EPDM/paraffin foams, degradation takes place in two different steps associated with the thermal degradation of paraffin and EPDM rubber. Moreover, the addition of paraffin leads to a decrease in thermal stability of the materials. Looking at the results summarized in Table 6, it is possible to observe that the T_5%_ values of the EPDM/paraffin foams are strongly lower than those of reference samples. For all paraffin amounts, a reduction of about 40% of T_5%_ is registered. Furthermore, the thermal degradation behavior of foams with the same paraffin content is quite similar, since the difference in T_peak1_ and T_peak2_ is very limited. On the other hand, for both production methods, a shift in T_peak1_ toward the corresponding value of neat RT70HC with an increasing amount of paraffin can be noticed. Considering the residue at 700 °C, it is possible to observe that, in all the cases, it decreases increasing the paraffin amount. Since neat paraffin completely degrades without leaving any solid residues, m_700_ in EPDM/paraffin foams is related to the presence of carbon black in the EPDM rubber. In fact, decreasing the EPDM amount in the foams leads to a decrease in m_700_, proportionally to the paraffin concentration. Finally, analyzing the effective paraffin content in the samples (PCM_eff_), it can be noticed that the weight percentages of paraffin are very similar to the theoretical values. This suggests that the PCM in the EPDM matrix is probably distributed homogeneously and that the production process is mild enough not to degrade the PCM.

### 3.5. DSC

**Figure 4:**
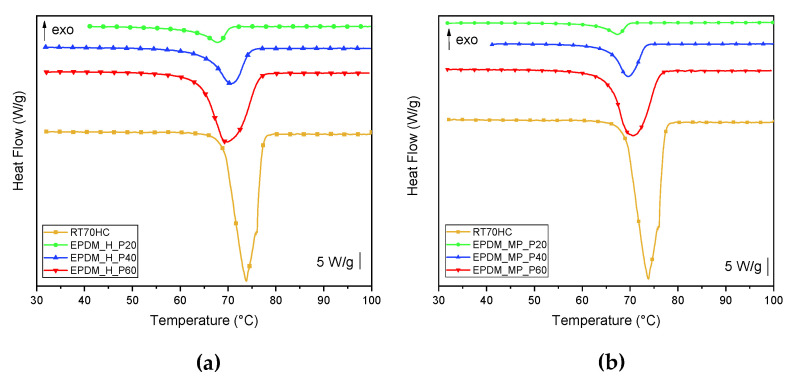
DSC thermograms (first heating scan) of neat paraffin (RT7OHC) and paraffin-filled foams produced with (**a**) H and (**b**) MP foaming agents.

To investigate the influence of paraffin amount and production process on thermal properties of prepared foams, DSC analysis has been carried out. DSC thermograms of neat paraffin and of the foams at different PCM contents are presented in Figure 4a,b, while the most significant results are reported in Table 7.

From thermograms reported in Figure 4a,b, it is possible to observe the melting and crystallization peaks of paraffin at a temperature around 70 °C. The intensity of the peak progressively increases with increasing paraffin amount, in a percentage that reflects the PCM content in the foam. Melting temperatures in the first and second heating scan, T_m1_ and T_m2_, are comparable to the ones of neat paraffin. On the other hand, the crystallization temperature (T_c_) in the foams is slightly lower in comparison to neat paraffin, and decreases from 67 °C to approx. 58 °C. This is probably due to the lower thermal conductivity of the foams. For as concerns Δ*H_m_* and Δ*H_c_* values, it is possible to notice that all the prepared foams show a good thermal energy storage capability, in both the heating and cooling scans. In addition, melting and crystallization enthalpy values are proportional to the PCM content in the foams. For example, Δ*H_m_*_2_ values for EPDM_H_P20 (47.8 J/g) and EPDM_MP_P20 (44.4 J/g) corresponds to 17.3% and 16.1% of Δ*H_m_*_2_ of neat RT70HC, respectively. Finally, by analyzing the effective paraffin contents in the first heating scan, cooling scan and second heating scan (i.e., *PCM_m_*_1_*^eff^*, *PCM_c_^eff^*, and *PCM_m_*_2_*^eff^*), it can be observed that these values are pretty similar and no significant differences are present in the three thermal ramps. Comparing these values with the effective PCM content obtained from TGA tests (*PCM_eff_*), reported in Table 6, it is clear that the effective content of paraffin in the foams obtained in DSC tests results to be lower with respect to that obtained from TGA analysis. As it was observed in our previous work [46], the confinement of the PCM in the EPDM matrix probably limits and makes crystallization of paraffin more difficult, leading so to a lower melting and crystallization enthalpy.

**Figure 5:**
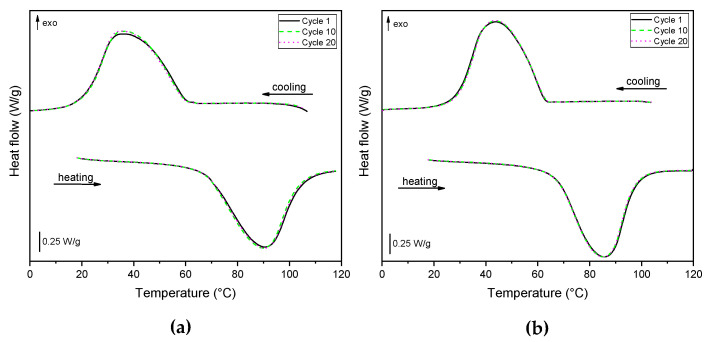
DSC thermograms of the 1st, 10th, and 20th cycles on (**a**) EPDM_H_P60 and (**b**) EPDM_MP_P60.

Cyclic DSC tests were performed on EPDM_H_P60 and EPDM_MP_P60 to evaluate the retention of the thermal energy storage/release effect over cyclic conditions. Figure 5a,b shows DSC thermograms of the 1st, 10th, and 20th thermal cycles of the selected formulations. The thermograms at different cycles are overlapped and no significant differences can be observed in the phase change temperature and enthalpies, suggesting that the thermal properties of EPDM/paraffin foams are stable up to 20 cycles.

### 3.6. Thermal Conductivity Measurements

**Figure 6:**
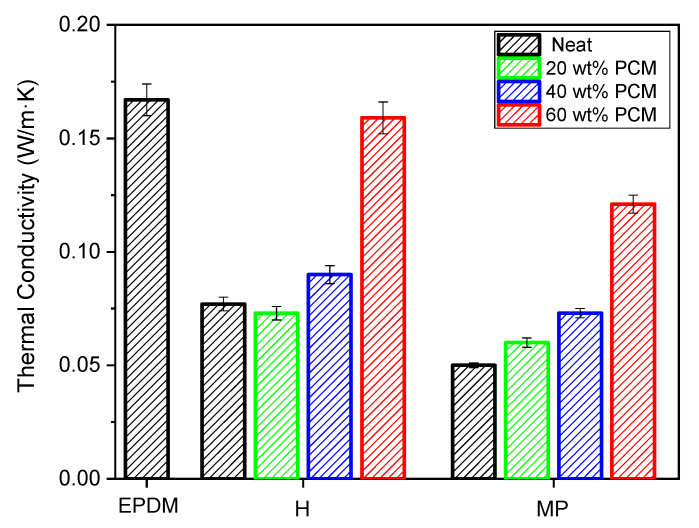
Results of thermal conductivity tests on bulk EPDM sample and on the prepared foams.

Thermal conductivity values of bulk EPDM sample and of the prepared foams are reported in Figure 6.

The thermal conductivity is strongly reduced moving from the unfoamed to foamed samples. In particular, a decrease of more than 50% for both the production processes can be highlighted. The lowest value of thermal conductivity, i.e., 0.05 W/m·K, is registered in EPDM_MP foam, in agreement to its closed-cell morphology evidenced in density measurement and SEM micrographs. Moreover, the addition of paraffin leads to an increase in thermal conductivity. This was expected, since ʎ of paraffin (0.2 W/m·K) is higher than the one of unfilled EPDM foams, and the total porosity significantly decreases with increasing PCM amount. However, the EPDM/paraffin foams present good thermo-insulating properties up to a paraffin content of 40 wt%.

### 3.7. Rheology

**Figure 7:**
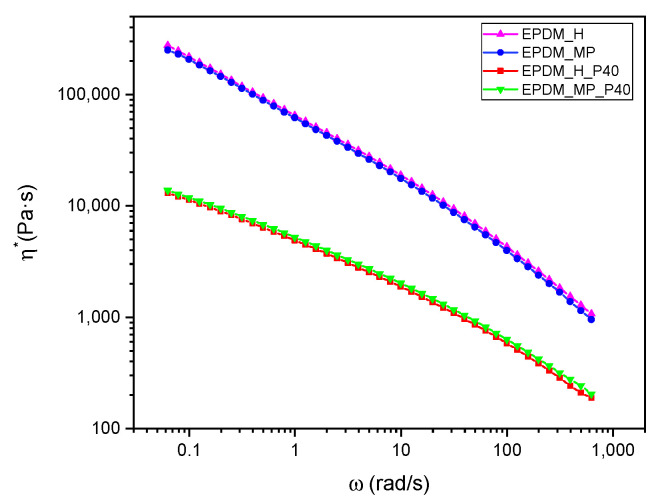
Dynamic rheological curves on the not-vulcanized EPDM mixtures. Complex viscosity (η*) as a function of angular frequency (ω) for EPDM_H, EPDM_MP, EPDM_H_40, and EPDM_MP_P40 samples.

Dynamic rheological tests were performed to investigate the effect of the foaming agent on the complex viscosity of the not vulcanized mixture. The frequency dependence of the complex viscosity for EPDM_H, EPDM_MP, EPDM_H_P40, and EPDM_MP_P40 un-vulcanized mixtures is reported in Figure 7.

Analyzing the results in Figure 7, the type of foaming agent clearly does not affect the complex viscosity of the system since η* curves nearly overlap for the same paraffin content. Consequently, the differences found in total porosity among the produced foams could be only related to the different working principles of H and MP foaming agents. On the other hand, paraffin has a strong influence on EPDM rubber viscosity at 100 °C. The addition of 40 wt% of PCM to the elastomeric compound leads to a decrease in η* of about one order of magnitude in the angular frequency range investigated. However, neat and paraffin-filled samples exhibit the same trend, characterized by a decrease in η* by increasing angular frequency. Finally, it must be underlined that the complex viscosity values of EPDM_H and EPDM_MP obtained in the range 0.1–103 rad/s are in agreement with values found in the literature [47].

### 3.8. Quasi-Static Tensile Tests

**Figure 8:**
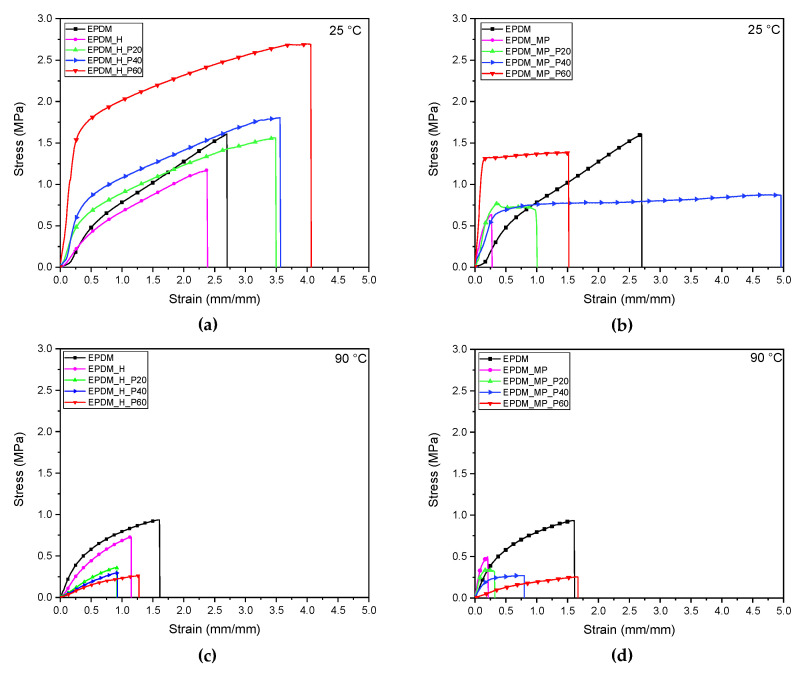
Representative tensile stress-strain curves at 25 °C and 90 °C of bulk EPDM sample and foams produced with (**a**,**c**) H and (**b**,**d**) MP foaming agents.

Quasi-static tensile tests were carried out at 25 °C and 90 °C, i.e., below and above the melting point of the PCM, in order to investigate the influence of the amount and physical state of paraffin on the mechanical properties of the foams. Stress-strain curves of neat EPDM samples and EPDM/paraffin foams are reported in Figure 8a–d, while the main results are summarized in Table 8.

Looking at Figure 8a–d, the different mechanical response shown by the neat foams at both the investigated temperatures can be highlighted. In particular, EPDM_MP sample is characterized by the highest elastic modulus (11.60 and 15.48 MPa·cm^3^/g at 25 and 90 °C, respectively) while EPDM_H presents the highest stress and strain at break, with a mechanical response similar to bulk EPDM. The high specific stiffness found in EPDM_MP sample, greater than bulk EPDM, could probably be attributed to the presence of microcapsules containing the physical blowing agent. Finally, by analyzing the results in Table 8, it is evident that the temperature, in the range explored, does not affect the mechanical properties of the neat samples in a significant way.

Focusing then on EPDM/paraffin foams, it can be observed that their mechanical properties at 25 °C are considerably higher than those obtained at 90 °C. This is due to the different effects exerted by liquid and solid paraffin in the samples. The results of the tests performed at 25 °C in Table 8 evidence an increase in elastic modulus with an increasing in PCM content in all the expanded rubbers. This suggests that the elastic modulus of paraffin at room temperature is higher than that of the EPDM matrix. The use of MP foaming agent leads to the production of stiffer foams with respect to H. Considering the stress and strain at break, different trends among the samples with increasing PCM content are observed. In particular, while in foams produced with H an increase both in σ_b_* and ε_b_ is registered, in the case of foams produced with MP, no clear trend can be found. Particular attention should be paid to EPDM_MP_P40 sample: a strain at break close to 5 mm/mm has been registered, considerably higher than ε_b_ of the other specimens. This result could be probably due to the non-homogeneous distribution of the PCM in this formulation. However, future analysis will be performed to confirm this hypothesis. From the results of the tests performed at 90 °C, presented in Table 8, it is clear that the addition of paraffin at the liquid state leads to a strong decrease of both elastic modulus and stress at break. On the other hand, the liquid PCM seems to act as a plasticizing agent within the material. In fact, an increment of ε_b_ can be evidenced by increasing the paraffin content.

The drop in E*, as a consequence of the PCM melting, suggests that the prepared EPDM/paraffin foams could potentially present a shape memory behavior. Future research will aim to investigate the shape memory performance of these expanded rubbers since such behavior could be desirable to improve and widen their application fields.

### 3.9. Quasi-Static Compression Tests

**Figure 9:**
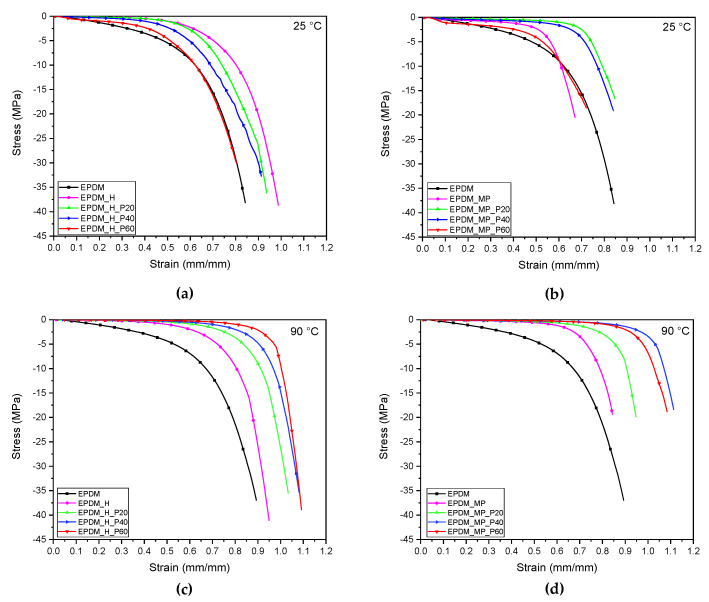
Representative compressive stress strain curves at 25 °C and 90 °C of bulk EPDM sample and foams produced with (**a**,**c**) H and (**b**,**d**) MP foaming agents.

Quasi-static compression tests were carried out at 25 °C and 90 °C, i.e., below and above the melting point of paraffin, in order to investigate the influence of the amount and physical state of paraffin on the compressive properties of the foams. Compressive curves of neat EPDM samples and EPDM/paraffin foams are reported in Figure 9a–d, while the main results are summarized in Table 9.

Analyzing at first the results related to neat EPDM samples, it is possible to highlight that compressive behavior of unfoamed and foamed samples is completely different, both at 25 °C and 90 °C. As shown in Figure 9a–d, bulk EPDM undergoes a rapid increase in stress, even at low deformation values, unlike the expanded samples whose curves show a large stress plateau at low strain levels. This stress plateau is due to the progressive deformation of the porosity as densification occurs, causing an increase in stress with strain at a certain strain value. The strain at which densification starts for EPDM_MP and EPDM_H samples is approx. 0.56 mm/mm and 0.71 mm/mm at 25 °C, respectively, and about 0.4 mm/mm for both types of foams at 90 °C. Elastic moduli of all foamed samples are lower than the modulus of bulk EPDM sample both at 25 °C and 90 °C. However, the temperature seems to slightly affect the moduli of neat samples, since E_c_* decreases moving from 25 °C to 90 °C, probably due to the softening of rubber. Values of σ_0.6_ are the highest for EPDM_MP and this behavior is probably caused by residual microcapsule parts, as was observed for the elastic modulus.

Moving to the analysis of the compressive behavior of EPDM/paraffin foams, at 25 °C the increase in paraffin concentration leads to an increase in compressive modulus. This is accompanied by a decrease in ε_0.5_, strain at which densification starts, and an increment of σ_0.6_*. In particular, the reduction in ε_0.5_ is visible looking at the plateau’s extension in the relative compressive curves. It decreases moving from a PCM content of 20 wt% to 60 wt% and this is correlated to the corresponding reduction in total porosity in the foams (*P_tot_*) previously evidenced. At 90 °C, the compressive modulus of all the investigated foams is almost half in comparisons to the values obtained at 25 °C due to the presence of liquid paraffin that act as a softener of the elastomeric matrix. This behavior can be observed by analyzing the curves presented in Figure 9c,d. The stress plateau is characterized by very low values of stress, which decreases by increasing the amount of PCM in the foams, as can be noticed by looking at σ_0.6_*. The plateau ends at deformation levels higher than those observed at room temperature, once again for the softening action of paraffin. In particular, densification occurs at a strain level in the range 0.43–0.70 mm/mm and 0.51–0.73 mm/mm in samples produced with H and MP, respectively.

### 3.10. Shore A

Finally, Shore A values at 25 and 90 °C of the bulk EPDM, neat paraffin, and all produced foams are reported in Table 10.

As expected, Shore A hardness reduces moving from bulk EPDM to neat foamed samples, both at 25 °C and 90 °C. Focusing on the values obtained at 25 °C, i.e., below the melting temperature of paraffin, it is clear that the PCM content strongly affects the hardness of the foams. In all the produced samples, the greater the paraffin content, the higher the hardness. Finally, from a practical point of view, it has to be underlined that higher hardness values are also synonymous with difficult deformability. On the other hand, values obtained at 90 °C, i.e., above the melting temperature of paraffin, show an opposite trend than those determined at 25 °C. In particular, for all the produced samples, a decrease in hardness with increasing paraffin amount has been registered.

## 4. Conclusions

In this work, the preparation and thermo-mechanical characterization of novel EPDM/paraffin foams have been reported. SEM micrographs and density measurements showed that the foaming agent Micropearl F82^®^ (MP, Lehvoss Italia s.r.l. Saronno, Italia) led to the formation of a closed-cell porosity, while Hostatron P0168^®^ (H, Clariant GmbH, Ahrensburg, Germany) of a mixed closed-cell/open-cell morphology. On the other hand, samples produced through salt leaching were mainly characterized by an open-cell porosity. For all the EPDM/paraffin foams, the total porosity decreased by increasing the paraffin amount. This experimental evidence was related to the action of the PCM to hinder the expansion of the samples produced with foaming agents and, in the case of expanded rubbers produced through salt leaching, to the filling of the open pores with increasing paraffin content. The qualitative analysis of paraffin leakage revealed that at 90 °C only the foams produced through salt leaching suffered from significant PCM leakage. Consequently, the thermo-mechanical properties were investigated only in samples produced with H and MP.

TGA results evidenced that the thermal degradation resistance of the studied foams decreased with the addition of paraffin but, for all PCM contents, it remained at values well above the operating temperature at which EPDM foams are usually employed. DSC analysis revealed that EPDM/paraffin foams were characterized by good thermal energy storage properties, especially at higher PCM contents. A more in-depth analysis of the TES capability of the foams will be the object of our future works. Thermal conductivity analysis evidenced an increase in thermal conductivity of the produced EPDM/paraffin foams with increasing PCM content. However, for all the types of samples, a value lower than 0.16 W/m·K was found, highlighting the good thermal insulation properties of the foams. In particular, the lowest thermal conductivity values were registered in samples produced with MP, which was expected thanks to its closed-cell morphology.

From quasi-static tensile tests at 25 °C, i.e., below the melting temperature of the PCM, the stiffness of the foams increased with the paraffin concentration, while the opposite was observed at 90 °C, when the paraffin was in the molten state. Such trends were confirmed also in quasi-static compression tests. These tests also showed that EPDM/paraffin foams presented a very large stress plateau due to the progressive deformation of the porosity.

In conclusion, this work demonstrated that, among the studied foams, only the ones produced with MP and H could be potentially used for thermal management applications in electronics. The results of the salt leaching technique, despite the environmental advantages, were not adequate as shape-stabilizers for paraffin with a melting temperature of 70 °C. Future research will aim to study more in-depth the TES properties of the foams and investigate if these expanded rubbers could exhibit a shape memory behavior.

## Figures and Tables

**Table 1:** Composition of the elastomeric compound used in this work.

Material	Quantity (phr)
Vistalon^®^ 2504	100
Sulfur (vulcanizing agent)	3.00
Zinc oxide (activator)	3.00
Stearic acid (activator)	1.00
Carbon black (reinforcing filler)	20.00
TMTD * (accelerator)	0.87
ZDBC ** (accelerator)	2.50

* TMTD = tetramethylthiuram disulfide; ** ZDBC = zinc dibutyldithiocarbamate.

**Table 2:** Compounding temperatures used for the prepared samples.

Sample	Compounding Temperature (°C)
Not expanded EPDM	40 ± 1
EPDM foams produced using foaming agents	40 ± 1
EPDM foams produced through salt leaching method	60 ± 1
EPDM/paraffin foams produced using foaming agents	70 ± 1
EPDM/paraffin foams produced through salt leaching method	60 ± 1

**Table 3:** List of the prepared samples and their nominal composition.

Sample	Vistalon 2504 [phr]	Hostatron P0168 [phr]	Micropearl F82 [phr]	NaCl [phr]	PEG [phr]	RT70HC [phr/wt%/vv%]
EPDM	100	-	-	-	-	-
EPDM_H	100	1.3	-	-	-	-
EPDM_H_P20	100	1.3	-	-	-	33.0/20.0/22.0
EPDM_H_P40	100	1.3	-	-	-	87.0/40.0/43.0
EPDM_H_P60	100	1.3	-	-	-	196.0/60.0/63.0
EPDM_MP	100	-	14	-	-	-
EPDM_MP_P20	100	-	14	-	-	33.0/20.0/22.0
EPDM_MP_P40	100	-	14	-	-	87.0/40.0/43.0
EPDM_MP_P60	100	-	14	-	-	196.0/60.0/63.0
EPDM_SL	100	-	-	305	30	-
EPDM_SL_P20	100	-	-	305	30	33.0/20.0/22.0
EPDM_SL_P40	100	-	-	305	30	87.0/40.0/43.0
EPDM_SL_P60	100	-	-	305	30	196.0/60.0/63.0

**Table 4:** Dimension of the pores of the produced foams.

Sample	Diameter of the Pores (µm)
EPDM_H	271 ± 150
EPDM_H_P20	254 ± 95
EPDM_H_P60	366 ± 306
EPDM_MP	110 ± 46
EPDM_MP_P20	82 ± 39
EPDM_MP_P60	64 ± 22
EPDM_SL	2 ± 1
EPDM_SL_P20	93 ± 63
EPDM_SL_P60	-

**Table 5:** Density and open, closed, and total porosity of the prepared foams.

Sample	*ρ_app_* (g/cm^3^)	*ρ_geom_* (g/cm^3^)	*ρ_bulk_* (g/cm^3^)	*OP* (vol%)	*CP* (vol%)	*P_tot_* (vol%)
EPDM	-	0.996 ± 0.02	0.996	-	-	-
EPDM_H	0.79 ± 0.03	0.61 ± 0.05	0.996	19.6 ± 9.1	18.9 ± 13.6	38.5 ± 4.6
EPDM_H_P20	0.72 ± 0.02	0.55 ± 0.02	0.97	23.5 ± 4.5	20.2 ± 6.1	43.7 ± 1.6
EPDM_H_P40	0.78 ± 0.03	0.66 ± 0.01	0.946	15.6 ± 4.6	14.6 ± 5.7	30.2 ± 1.1
EPDM_H_P60	0.80 ± 0.02	0.70 ± 0.01	0.923	12.6 ± 3.5	11.9 ± 4.6	24.5 ± 1.1
EPDM_MP	0.30 ± 0.01	0.25 ± 0.01	0.996	17.0 ± 5.7	57.7 ± 6.6	74.7 ± 1.0
EPDM_MP_P20	0.37 ± 0.01	0.30 ± 0.02	0.97	18.9 ± 6.1	50.3 ± 7.8	69.3 ± 1.7
EPDM_MP_P40	0.44 ± 0.01	0.37 ± 0.02	0.946	16.9 ± 7.3	44.1 ± 9.6	61.0 ± 2.2
EPDM_MP_P60	0.76 ± 0.02	0.56 ± 0.02	0.923	26.2 ± 4.0	13.1 ± 5.7	39.3 ± 1.8
EPDM_SL	0.98 ± 0.03	0.35 ± 0.02	0.996	64.3 ± 3.1	0.6 ± 5.2	64.9 ± 2.1
EPDM_SL_P20	0.95 ± 0.01	0.67 ± 0.02	0.97	29.2 ± 2.6	1.6 ± 5.0	30.8 ± 2.2

*ρ_app_* = apparent density from pycnometry; *ρ_geom_* = geometrical density; *ρ_bulk_* = bulk density; *OP* = open porosity; *CP* = closed porosity; *P_tot_* = total porosity.

**Table 6:** Results of the TGA tests on bulk EPDM sample, neat paraffin (RT70HC), and the prepared foams.

Sample	T_5%_ (°C)	T_peak1_ (°C)	T_peak2_ (°C)	m_700_ (%)	PCM_eff_ (%)
EPDM	420.2	-	484.4	19.9	-
RT70HC	258.7	338.3	-	0.0	100.0
EPDM_H	402.8	-	481.0	19.2	-
EPDM_H_P20	246.5	281.8	478.6	15.6	23.5
EPDM_H_P40	250.8	308.6	479.9	11.6	40.9
EPDM_H_P60	253.2	318.6	481.7	8.9	58.5
EPDM_MP	317.3	-	487.5	20.7	-
EPDM_MP_P20	252.0	311.7	487.8	16.8	25.0
EPDM_MP_P40	254.5	313.1	491.6	13.2	42.2
EPDM_MP_P60	250.2	332.8	488.6	9.3	60.8

T_5%_ = temperature corresponding to a mass loss of 5%; T_peak1_ = temperature of the maximum degradation rate of RT70HC; T_peak2_ = temperature of the maximum degradation rate of EPDM rubber; m_700_ = mass residue at 700 °C; PCM_eff_ = effective paraffin content (calculated from the mass loss at 400 °C).

**Table 7:** Results of the DSC tests on neat paraffin and on the prepared foams.

Sample	T_m1_ (°C)	Δ*H_m_*_1_ (J/g)	T_c_ (°C)	Δ*H_c_* (J/g)	T_m2_ (°C)	Δ*H_m_*_2_ (J/g)	*PCM_m_*_1_*^eff^* (%)	*PCM_c_^eff^* (%)	*PCM_m_*_2_*^eff^* (%)
RT70HC	68.4	277.4	66.9	274.2	69.4	276.1	-	-	-
EPDM_H_P20	67.8	47.3	58.6	47.5	68.0	47.8	17.1	17.3	17.3
EPDM_H_P40	70.6	94.8	57.6	94.1	70.3	94.2	34.2	34.3	34.1
EPDM_H_P60	70.0	144.6	62.8	143.6	70.5	145.4	52.1	52.4	52.7
EPDM_MP_P20	67.4	44.2	58.9	40.1	67.7	44.4	15.9	14.6	16.1
EPDM_MP_P40	69.9	93.5	59.6	89.5	69.4	96.1	33.7	32.6	34.8
EPDM_MP_P60	70.6	145.8	63.7	145.2	70.3	145.7	52.6	52.9	52.8

T_m1_ = melting temperature in 1st heating scan; *ΔH_m_*_1_ = melting enthalpy in 1st heating scan; T_c_ = crystallization temperature; *ΔH_c_* = crystallization enthalpy; T_m2_ = melting temperature in 2nd heating scan; *ΔH_m_*_2_ = melting enthalpy in 2nd heating scan; *PCM_m_*_1_*^eff^* = PCM content in 1st heating scan; *PCM_c_^eff^* = PCM content in cooling scan; *PCM_m_*_2_*^eff^* = PCM content in 2nd heating scan.

**Table 8:** Results of quasi-static tensile tests at 25 °C on the prepared samples.

		25 °C			90 °C	
Sample	E* (MPa·cm^3^/g)	σ_b_* (MPa·cm^3^/g)	ε_b_ (mm/mm)	E* (MPa·cm^3^/g)	σ_b_* (MPa·cm^3^/g)	ε_b_ (mm/mm)
EPDM	1.35 ± 0.09	1.58 ± 0.15	3.00 ± 0.55	1.56 ± 0.20	0.90 ± 0.09	1.42 ± 0.20
EPDM_H	1.21 ± 0.39	1.21 ± 0.13	2.53 ± 0.27	1.37 ± 0.08	1.19 ± 0.08	1.03 ± 0.17
EPDM_H_P20	3.18 ± 0.98	1.63 ± 0.31	3.77 ± 0.62	0.91 ± 0.06	0.68 ± 0.03	0.88 ± 0.03
EPDM_H_P40	4.42 ± 0.42	1.99 ± 0.35	4.71 ± 1.73	0.58 ± 0.02	0.46 ± 0.05	0.97 ± 0.17
EPDM_H_P60	7.96 ± 2.18	2.57 ± 0.60	4.15 ± 1.42	0.41 ± 0.03	0.40 ± 0.04	1.28 ± 0.17
EPDM_MP	11.60 ± 0.86	2.61 ± 0.04	0.28 ± 0.03	15.48 ± 0.45	1.90 ± 0.06	0.19 ± 0.05
EPDM_MP_P20	12.97 ± 0.92	2.45 ± 0.09	0.47 ± 0.22	10.54 ± 0.77	1.28 ± 0.08	0.30 ± 0.06
EPDM_MP_P40	12.08 ± 0.39	2.56 ± 0.06	4.26 ± 2.30	2.82 ± 0.11	0.71 ± 0.01	0.92 ± 0.25
EPDM_MP_P60	17.42 ± 2.18	2.60 ± 0.20	1.82 ± 0.57	0.33 ± 0.01	0.39 ± 0.04	1.48 ± 0.21

E* = normalized elastic modulus; σ_b_* = normalized stress at break; ε_b_ = strain at break.

**Table 9:** Results of the quasi-static compression tests on the prepared samples.

		25 °C			90 °C	
Sample	E_c_* (MPa cm^3^/g)	σ_0.6_* (MPa cm^3^/g)	ε_5_ (mm/mm)	E_c_* (MPa cm^3^/g)	σ_0.6_* (MPa cm^3^/g)	ε_5_ (mm/mm)
EPDM	9.31 ± 0.94	8.86 ± 1.99	0.49 ± 0.04	7.43 ± 0.62	6.70 ± 1.27	0.13 ± 0.03
EPDM_H	1.41 ± 0.54	3.55 ± 1.18	0.71 ± 0.05	2.15 ± 0.31	3.25 ± 0.93	0.38 ± 0.02
EPDM_H_P20	2.02 ± 0.46	4.90 ± 3.09	0.69 ± 0.08	0.37 ± 0.14	0.79 ± 0.40	0.63 ± 0.07
EPDM_H_P40	5.71 ± 2.97	9.02 ± 3.91	0.58 ± 0.06	0.67 ± 0.05	0.74 ± 0.14	0.43 ± 0.37
EPDM_H_P60	7.55 ± 1.69	12.78 ± 2.23	0.52 ± 0.03	0.61 ± 0.24	0.51 ± 0.16	0.70 ± 0.09
EPDM_MP	7.53 ± 1.03	41.98 ± 22.53	0.56 ± 0.05	3.47 ± 0.90	20.9 ± 38.65	0.42 ± 0.18
EPDM_MP_P20	3.00 ± 0.31	3.67 ± 0.44	0.75 ± 0.02	1.82 ± 0.13	2.00 ± 0.25	0.56 ± 0.01
EPDM_MP_P40	3.98 ± 0.57	4.81 ± 0.79	0.71 ± 0.03	0.89 ± 0.06	0.82 ± 0.04	0.73 ± 0.02
EPDM_MP_P60	5.93 ± 0.76	15.12 ± 2.65	0.54 ± 0.03	0.66 ± 0.04	0.73 ± 0.03	0.51 ± 0.05

E_c_* = normalized compression modulus; σ_0.6_* = normalized stress at strain equal to 0.6 mm/mm; ε_5_ = strain at 5 MPa.

**Table 10:** Shore A values.

Sample	Shore A at 25 °C	Shore A at 90 °C
EPDM	58 ± 2	59 ± 1
EPDM_H	34 ± 3	32 ± 1
EPDM_H_P20	47 ± 3	27 ± 1
EPDM_H_P40	56 ± 2	23 ± 2
EPDM_H_P60	69 ± 9	18 ± 5
EPDM_MP	47 ± 1	46 ± 2
EPDM_MP_P20	48 ± 3	39 ± 3
EPDM_MP_P40	56 ± 1	20 ± 1
EPDM_MP_P60	72 ± 2	15 ± 1

## Data Availability

Data available on request.
